# Mutagenesis of DsbAss is Crucial for the Signal Recognition Particle Mechanism in *Escherichia coli*: Insights from Molecular Dynamics Simulations

**DOI:** 10.3390/biom9040133

**Published:** 2019-04-03

**Authors:** Faiza Gul Durrani, Roquyya Gul, Muhammad Usman Mirza, Naheed Nazly Kaderbhai, Matheus Froeyen, Mahjabeen Saleem

**Affiliations:** 1 School of Biological Sciences, Quaid-e-Azam Campus, University of the Punjab, Opp. Sheikh Zaid Hospital, Canal Bank Road, Lahore 54590, Pakistan; faiza_phd@hotmail.com; 2 Faculty of Biological Sciences, Gulab Devi Educational Complex, Lahore, Ferozpur Road, Lahore 54000, Pakistan; 3 Department of Pharmaceutical and Pharmacological Sciences, Rega Institute for Medical Research, Medicinal Chemistry, University of Leuven, B-3000 Leuven, Belgium; muhammadusman.mirza@kuleuven.be (M.U.M.); mathy.froeyen@rega.kuleuven.be (M.F.); 4 Center for Research in Molecular Medicine, The University of Lahore, Lahore 54000, Pakistan; 5 Department Institute of Biological, Environmental and Rural Sciences, Aberystwyth University, Penglais, Aberystwyth, Ceredigion SY23 3FL, UK; nnk@aber.ac.uk; 6 Institute of Biochemistry and Biotechnology, Quaid-e-Azam Campus, University of the Punjab, Opp. Sheikh Zaid Hospital, Canal Bank Road, Lahore 54590, Pakistan; mahjabeen.ibb@pu.edu.pk

**Keywords:** DsbA signal sequence, ovine growth hormone, signal recognition particle system, molecular dynamics simulation, molecular mechanics generalized born surface area

## Abstract

The disulfide bond signal sequence (DsbAss) protein is characterized as an important virulence factor in gram-negative bacteria. This study aimed to analyze the “alanine” alteration in the hydrophobic (H) region of DsbAss and to understand the conformational DsbAss alteration(s) inside the fifty-four homolog (Ffh)-binding groove which were revealed to be crucial for translocation of ovine growth hormone (OGH) to the periplasmic space in *Escherichia coli* via the secretory (Sec) pathway. An experimental design was used to explore the hydrophobicity and alteration of alanine (Ala) to isoleucine (Ile) in the tripartite structure of DsbAss. As a result, two DsbAss mutants (Ala at positions -11 and -13) with same hydrophobicity of 1.539 led to the conflicting translocation of the active OGH gene. We performed molecular dynamics (MD) simulations and molecular mechanics generalized born surface area (MM-GBSA) binding free energy calculations to examine the interaction energetic and dynamic aspects of DsbAss/signal repetition particle 54 (SRP54) binding, which has a principle role in *Escherichia coli* Sec pathways. Although both DsbAss mutants retained helicity, the MD simulation analysis evidenced that altering Ala-13 changed the orientation of the signal peptide in the Ffh M binding domain groove, favored more stable interaction energies (MM-GBSA ΔG_total_ = −140.62 kcal mol^−1^), and hampered the process of OGH translocation, while Ala-11 pointed outward due to unstable conformation and less binding energy (ΔG_total_ = −124.24 kcal mol^−1^). Here we report the dynamic behavior of change of “alanine” in the H-domain of DsbAss which affects the process of translocation of OGH, where MD simulation and MM-GBSA can be useful initial tools to investigate the virulence of bacteria.

## 1. Introduction

Almost all previously easily treatable infections have developed antibiotic resistance, leading to “multidrug-resistant pathogens” [1,2,3]. This alarming situation needs a rapid solution as mortality rates by common infections caused by these multidrug-resistant pathogens will soon be much higher than those of the most feared disease, cancer [4]. To solve this dilemma, scientists have started developing an approach to destroying the virulence factor rather than bacterial growth [5,6]. From studies on a wide range of bacteria, the importance of “disulfide bond (Dsb) oxidative folding machinery” has emerged as the main area of interest [7,8]. The chemical structure of all the virulence factor proteins represents the correct positioning of the disulfide bond which activates the virulence factor [9]. 

The “Dsb” protein family has been comprehensively characterized, and type “DsbA” was considered as strongly oxidizing [10], and significantly important in the initiation process [7,11,12]. The occurrence of DsbA was found in almost all classes of Chlamydiales and Proteobacteria [7,8], and its absence showed incorrect folding of periplasmic and outer membrane proteins [13,14]. Furthermore, these bacteria showed functional changes in their virulence; toxins and motility were diminished when tested in animal models [7,8]. For example, *Vibrio cholerae* in the absence of DsbA caused low levels of cholera, enteropathogenic *Escherichia coli* with DsbA mutant showed low heat lability, and *Bordetella pertussis* secreted low levels of pertussis toxins [15,16]. The DsbA protein system has a central role inside the cell “periplasmic space” where it performs oxidation–reduction processing of cysteine residues to form disulfide bonds for a protein of interest. In eubacteria, the cleavable DsbAss works with signal recognition particle (SRP) upon binding with the fifty-four homolog (Ffh) region of SRP, which directs the rapid co-translational export of many proteins in the periplasmic space [17,18,19]. Various studies reported that the increased hydrophobicity of DsbAss directs efficient export of passenger proteins to the bacterial periplasm via the SRP targeting mechanism [20,21,22], and likewise in *E. coli* expression systems [23,24,25,26]. Taking into account hydrophobicity, we developed an alanine to isoleucine mutagenesis strategy in the DsbAss to analyze its impact on the translocation of the targeted protein, i.e., recombinant ovine growth hormone (rOGH).

We hypothesized that a new strategy could hamper the overall translocation process to study the impact of mutations in the hydrophobic core of DsbAss, rather than finding DsbA inhibitors to observe its effects on translocation of rOGH. The rOGH is a 22-kDa protein consisting of two disulfide bonds, and has been successfully expressed in a soluble form in high yield and translocated across periplasm of *E. coli* by DsbAss and the T7 promoter system [27]. The SRP targeting mechanism in *E. coli* [19] constitutes a “fifty-four homolog” protein (Ffh, a homolog of eukaryotic SRP54 protein), necessary for viability, efficient protein export, and 4.5S RNA. The primary sequence of Ffh is divided into three domains: the N-domain residing at the amino terminus; G-domain, which harbors the GTPase activity, and a methionine-rich M-domain involved with SRP54 binding of both RNA and the signal sequence [28]. The binding affinity of the Ffh/signal sequence increases with the hydrophobicity of the H-region of signal sequences [22,24]. It has been suggested that the magnitude of the interaction between SRP and the signal sequence correlates with the translocation efficiency as precursors with a more hydrophobic signal sequence are transported more efficiently [20,24]. The Ffh structural insight gives an adequate understanding of the signal sequence recognition mechanism via SRP [20,29]. The M-domain of SRP54, through a series of functional and cross-linking studies [30,31,32,33,34], has been identified to comprise of the signal sequence binding site [35,36,37]. Successive events during the SRP cycle require a rearrangement of the relative position of the N-, G-, and M-domains [37,38]. The binding of the signal sequence within M-domain induces conformational changes which stimulate the rotation of N-, G-domain to bring the GTPase domain closer, proceeding with the translocation process [36,39]. This mechanism has already been confirmed in various studies [29,36,39,40,41]. Recent successes towards the understanding of protein structural dynamics, which are intrinsic to biological processes, showed molecular simulation to be an efficient tool in investigating all atomic characterizations of biomolecular processes such as conformational transitions linked to protein function [42,43,44]. It was also useful for understanding the dynamic determinants of protein-peptide recognition [45], and recently, interactions with biological membranes [46].

The current study aimed to discover the structural insights of signal peptide (DsbAss) conformation inside the Ffh-binding groove by using MD simulations and MM-GBSA binding free energy calculations. Additionally, we identified the significant residues with favorable interaction energies through per-residue decomposition analysis of important amino acids lining the hydrophobic binding groove. Here, we report the dynamic behavior of change of “alanine” in H-domain of DsbA in affecting the process of translocation of rOGH, in good agreement with the experimental data. 

## 2. Materials and Methods 

### 2.1. E. Coli Strain, Plasmid, Enzymes, and Kits 

For expression studies, *E. coli* strain BL21 codon plus (DE3)-RIPL competent cells (Stratagene, CA, USA), and pET22b expression plasmid (Novagen Inc, Kenilworth, NJ, USA) were attained. To isolate the total RNA from the pituitary gland, Trizol reagent was purchased from Invitrogen. A QIAquick DNA extraction kit from agarose gel and QIAprep Spin Miniprep plasmid extraction kit were obtained from QIAGEN. From MBI Fermentas, InsT/A cloning kit, MMLV-Reverse Transcriptase, *Taq* DNA polymerase, restriction enzymes, IPTG, and T4 DNA ligase were procured. Polyclonal rabbit anti-recombinant caprine growth hormone (anti-rcGH) antibody was attained from in the house production facility at the School of Biological Sciences, University of the Punjab, Lahore, Pakistan.

### 2.2. Isolation of Total RNA and Construction of Recombinant pTzOGH-1 Plasmid

From pituitary gland of local ovine breed “LOHI”, total RNA was isolated by utilizing Trizol reagent. On the basis of the OGH sequence, forward primer (FP); FP-1 (5′-ATC CAT GGC CTT CCC AGC CAT G-3′) and reverse primer (RP); RP-1 (5′-TAG GAT CCG CAA CTA GAA GGC AGC-3′) were designed comprising *Nde*I and *Bam*HI restriction sites at the 5’end terminal. By using the above set of primers and isolated total RNA, a reverse transcription polymerase chain reaction was performed and complementary DNA (cDNA) was synthesized in a Fermentas Biosystems 2720 thermal cycler. For PCR amplification, denaturation, annealing, and extensions at 94, 65, and 72 °C, respectively, with a hold time of 1 min each for the 28 cycles, were executed. Further, the purified OGH amplicon was T/A cloned in pTz57R/T cloning vector to generate the recombinant pTzOGH-1 construct. 

### 2.3. Construction of Recombinant pOGH-1 to -8 Expression Plasmids

To study the expression and proper translocation of OGH into the extra-cytoplasmic space of *E. coli*, eight recombinant expression plasmids (pOGH-1 to -8) were constructed. These eight constructs were generated on the basis of upstream amino acid variations in the DsbAss. Eight FP (FP-1 to FP-8) were designed, with the *Nde*I site at the 5′-end, 57 nucleotides of the DsbAss (underlined), and six nucleotides of the OGH gene (bold), as shown in Table 1. FP-1 had native DsbAss. However, FP-2 to FP-8 were designed with a change of amino acid in the H-, C-, and N-domains of DsbAss (Table 1). Forward primers -1 to -8 and only one reverse primer, i.e., RP1 (5′-TAG GAT CCG CAA CTA GAA GGC AGC-3′) with the *Bam*HI site at the 5′-end were used to amplify eight OGH amplicons from pTzOGH-1 cloning vector. To generate a series of recombinant expression plasmids (pOGH-1 to -8), all the eight amplicons and the pET22b expression vector were restricted (*Nde*I/*Bam*HI), ligated, and primary cloned in the *E. coli* strain, DH5α, and then into the expression strain, BL21 codon plus.

### 2.4. Expression and Subcellular Fractionation of pOGH-1 to -8 Constructs

From all of the eight pOGH constructs, a single colony of each of the transformant was inoculated in Luria Broth (LB) medium (100 μg/mL ampicillin) and incubated overnight at 37 °C in an orbital shaker with a 150 rpm shake rate. Next morning, 1% (v/v) of an overnight culture for each construct was used to inoculate in 100 mL of LB medium. At 0.5 OD_600,_ 10 µM IPTG were induced in each of the constructs and at 6 h post induction, cells were collected by centrifugation at 5000 ×*g* for 5 min. The harvested cells were resuspended in 20 mL of STE (Sucrose Tris EDTA) buffer (20% v/v) sucrose in 0.33 M Tris-HCl and 1 mM EDTA (pH 8.0), and left for 10 min at room temperature. Then cells were centrifuged for 10 min at 4 °C at 7500 ×*g*. The supernatant was discarded while leaving behind approximately 250 µL volume to initially resuspend the cells and the proteins in the periplasm were extracted by rapid addition of 10 mL of chilled 0.5 mM MgCl_2_ using osmotic shock [47]. Further, the cell suspension was left on ice for 10 min and the periplasmic fraction was collected after centrifugation at 4 °C for 5 min at 5000 ×*g*. The cell pellet was processed for the cytoplasmic and membrane fractions by re-suspending in 20 mL TE buffer, 0.3 M Tris-HCl, and 1 mM EDTA (pH 8.0). The suspended cells were further sonicated by six bursts of 30 s each with a 3 min interval and centrifuged for 20 min at 4 °C at 5000 ×*g*. The collected supernatant was said to be the cytoplasmic fraction while the pellet was washed with 5 mL 0.3 M sucrose and 0.3 M Tris-HCl (pH 8.0), and centrifuged for 10 min at 11,000 ×*g*. Further, the supernatant was ultra-centrifuged for 1 h at 100,000 ×*g,* and the membrane pellet was finally resuspended in 5 mL of 0.3 M Tris-HCl (pH 8.0) [47].

### 2.5. Protein and Western Blot Analysis

Protein analysis of all the lysates was carried out on 15% SDS-PAGE as illustrated by Laemmli [48], and to visualize the resolved proteins, Coomassie brilliant blue R250 was used for staining. For the detection of the relative amount of protein in the bands and to quantify, a calibrated BioRad GS-800 densitometer and Phoretix 1D software (version 5.1) operating under MS Windows XPTM were utilized, respectively. Furthermore, to estimate the protein content spectrophotometrically at 595 nm with Coomassie Blue G-250 dye-binding procedure, Bradford assay [49] was performed, using bovine serum albumin (BSA) as the standard. Western blot analysis was carried out for the confirmation of the membrane-bound rOGH protein, using polyclonal rabbit anti-rcGH antibody derived from recombinant caprine GH which confirmed it as GH.

### 2.6. Molecular Modeling Studies

The Ffh M-domain of *E. coli* was retrieved from Protein Data bank under PDB ID: 1HQ1 (Resolution: 1.52, R-Free: 0.199) [50]. The co-crystallized structure with RNA was carefully investigated and revealed the finger loop. A 28 amino acid residue segment of the M-domain containing the proposed signal peptide recognition site was disordered in the crystals and was not observed in the electron density map. Therefore, homology modeling was performed for this segment by taking Ffh SRP54 domains from different organisms: *Bacillus subtilis* (*B. subtilis*) (PDB ID: 4UE4), *Thermus aquaticus* (*T. aquaticus*) (PDB ID: 2FFH), and *Methanocaldococcus jannaschii* (*M. jannaschii*) (PDB ID: 4XCO). Homology detection and structure prediction by HMM-HMM comparison (HHpred) was employed to search for possible templates, and multi-template homology modeling was performed by using MODELLER v9.15 [51,52]. The stereochemical assessment of the modelled loop and residue-by-residue geometry were checked by PROCHECK [53] and Molprobity [54]. Due to the flexibility of finger loop, the modeled M-domain was executed through 20 ns MD simulations using AMBER 16 to optimize a most stable conformation using root mean square deviation (RMSD) clustering. In order to analyze the DsbAss conformation inside signal peptide groove, protein molecular docking protocols were employed to obtain the most favorable conformation followed by MD simulations. Patch dock [55] with rescoring algorithm Fire dock server [56] and ClusPro 2.0 [57] were used for molecular docking. Input parameters for the Patch dock tool were PDB 3D coordinate files with default parameters. To evaluate each candidate transformation, the scoring function was used that was considered to have both atomic desolvation energy and geometric fit. Finally, clustering based on RMSD was applied to each solution to discard the redundant solution.

ClusPro 2.0 is the first fully automated and top-ranked performance in the latest rounds of CAPRI experiments [58] and evaluates the putative structure based on good electrostatic and desolvation free energies for clustering and rank them accordingly. ClusPro server produces four categories of predictive models, which are ranked by cluster size, including balanced, electrostatic-favored, hydrophobic-favored, and van der Waals + electrostatic (vdW + Elec). For current work, saturated clusters of best models were focused in all categories. The binding energies were calculated, and protein complexes of Ffh M-domain with all DsbAss were visually inspected for binding site residues and molecular interactions using UCSF Chimera v10 [59]. Binding site residues were defined as those residues with at least one heavy atom within a 4 Å distance from any heavy atom of the binding groove. To check the role in changing Ile to Ala at positions -11 and -13 in pOGH-2 and pOGH-5, respectively, extensive MD simulations of 200 ns were carried out to better comprehend the conformational stability of the DsbAss in signal peptide binding groove. For a positive control in MD simulations, study was corroborated with two reference structures, the co-crystalized conformation of signal sequence bound to the Ffh M-domain of *M. jannaschii* (PDB ID: 3NDB) [60], and after modelling of the missing finger loop in 3NDB (the same homology modeling procedure was implied as for Ffh M-domain of *E.coli*). Both sequences showed ≈40% identity in the corresponding Ffh M-domains.

### 2.7. Molecular Dynamics Simulation Protocol

All simulations were performed using the AMBER 16 simulation package [61]. We used the same MD simulation protocol described in our previous studies [62,63]. Briefly, the simulation system was prepared using the tleap modules of AMBER. An octahedral box filled with neutralizing Na+ ions and explicit TIP3P water molecules [64], extending 10.0 Å around the solute was utilized for the simulations. Particle-mesh Ewald electrostatics [65], periodic boundary conditions, and 9 Å cut-off were applied for non-bounded interactions. The SHAKE algorithm [66] together with a time step of 1 fs (for Langevin dynamics only during equilibration) or 2 fs was used to constrain the bonds to hydrogen atoms. A constant pressure (1 bar) and temperature (300 K) were maintained during 200 ns production simulations. The coupling constant for pressure was 2.0 ps while that for temperature was 5.0. Conjugate gradient method (subsequent 190 iterations) along with the steepest descent (first 10 iterations) was employed for energy minimization. This gradually reduced the restraint force constant from 10 to 0 kcal mol^−1^ Å2 on the protein atoms. Stepwise system equilibration was performed: (1) using 10 ps heating of the system with a Langevin thermostat (collision frequency γ = 1.0 ps^−1^) from 10 to 300 K, and restraint force (5 kcal mol^−1^ Å2), with both the volumes kept constant on the protein atom positions; (2) using a variation of the first step carried out prior to 20 ps heating of the system without any positional restraints; (3) using a constant volume and Langevin thermostat (γ = 0.5 ps^−1^), whereby a 20 ps MD simulation without positional restraints was performed at 300 K; (3) using constant pressure of 1.0 bar (coupling constant for pressure = 1.0 ps) with a Langevin thermostat (γ = 0.5 ps^−1^), maintained for 50 ps MD at 300 K without any restraints on the protein; and (5) using a 400 ps MD simulation at constant pressure of 1 bar (coupling constant for pressure = 2.0 ps and temperature = 5.0 ps) at 300 K and positional restraints. The cpptraj module of AMBER 16 [67] was utilized for the trajectory analysis.

### 2.8. MM-GBSA Binding Free Energy Calculations

The binding free energy (ΔG_tol_) of pOGH-2 and pOGH-5 complexes along with Ffh M-domain of *M. jannaschii* with bound signal sequence (with or without finger loop) as a positive control were calculated through MM-GBSA module of AMBER 16. The details of the MM-GBSA method have been explained elsewhere [68,69]. The snapshots from last 20 ns MD trajectory of the complex with an interval of 2.0 ps were generated, and total binding free energy (ΔG_tol_) was calculated, which is the sum of molecular mechanics energy (ΔE_MM_) and solvation free energy (ΔG_sol_). Both ΔE_MM_ and ΔG_sol_ are further divided into internal energy (ΔE_int_), electrostatic energy (ΔE_ele_), and van der Waals (ΔE_vdw_) energy in the gas phase, and polar (ΔG_p_) and non-polar (ΔG_np_) contributions to the solvation free energy as follows:

(1) $$\Delta E_{\mathrm{MM}}=\Delta E_{\mathrm{int}}+\Delta E_{\mathrm{ele}}+\Delta E_{\mathrm{vdw}}$$

(2) $$\Delta G_{\mathrm{sol}}=\Delta G_{p}+\Delta G_{\mathrm{np}}$$

(3) $$\Delta G_{\mathrm{tol}}=\Delta E_{\mathrm{MM}}+\Delta G_{\mathrm{sol}}$$

Normal mode analysis (nmode module) of AMBER 16 [61] was performed with the MM-GBSA method to calculate the contributions of each residue of the binding groove to the total binding free energy.

## 3. Results

### 3.1. rOGH Expression Analysis by Mutant Signal Sequences

**Figure 1:**
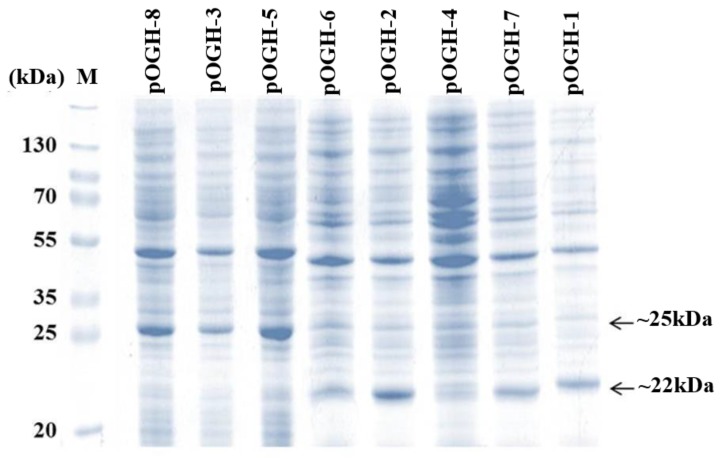
Total cell protein analysis of *Escherichia coli* transformed with pOGH-1 to -8 constructs on 15% SDS gels. M represents a molecular weight marker. pOGH-1 to -8 show expression of recombinant ovine growth hormone (rOGH) in eight constructs.

The DsbA mutant constructs with the OGH gene (pOGH-1 to -8) were transformed into *E. coli* BL21 (DE3) codon plus cells and grown in LB–ampicillin medium to an OD_600_ of 1.2 as explained in Materials and Methods. The protein expression of each construct based on OD_600_ values was analyzed by 15% SDS-PAGE. The calculated molecular weight of rOGH was ≈22 kDa, but a varying trend in rOGH mass and relative expression level was seen in different constructs of pOGH 1-8 (Figure 1). As shown, the pOGH constructs -1, -2, -6 and -7 showed bands of rOGH at the expected position, i.e., ≈22 kDa on the SDS gel, whereas pOGH-3, -5- and -8 showed bands at positions a little higher (≈25 kDa) than the calculated molecular mass of rOGH. Since the expected size of DsbAss was ≈2 kDa, the higher molecular mass appeared on SDS-PAGE gels might be the reason for incomplete processing of DsbAss during the process of translocation. The construct pOGH-4 appeared to express the rOGH ≈22 kDa with a barely detectable expression as confirmed by Western blot analysis (data not shown).

### 3.2. Impact of Ala Substitutions in DsbAss H-Domain

The amino acid structure of DsbA was composed of 28%, i.e., five alanine residues, in an 18 amino acid (KKIWLALAGLVLAFSASA)-long structure. The hydrophobic domain of DsbA constitutes most of it at positions −3, −6, −11, and −13 with respect to the signal peptidase cleavage site (Table 2). We targeted these Ala residues in all three parts of the DsbA structure; thus constructs of pOGH-2 to pOGH-6 had Ile at respective positions of Ala (Table 2). The expression levels of rOGH in these constructs ranged from undetectable (rOGH-4) to ≈25% (rOGH-5) of the total *E. coli* cellular proteins when observed on SDS-PAGE. The variation of molecular mass was also seen in the case of rOGH-5 and -3 (≈25 kDa) and rOGH-2, -4, and -6 proteins (≈22 kDa). The Swiss ExPASy Protparam tool was utilized to calculate the hydropathy index of each pOGH construct (Table 2). Among all variants, pOGH-2 and pOGH-5 were based on the variation of single Ala in the hydrophobic region at position -11 and -13 and revealed same hydropathy index i.e. 1.539, while the molecular mass of the protein in the SDS-PAGE was ≈22 kDa for pOGH-2 and ≈25 kDa for pOGH-5.

**Figure 2:**
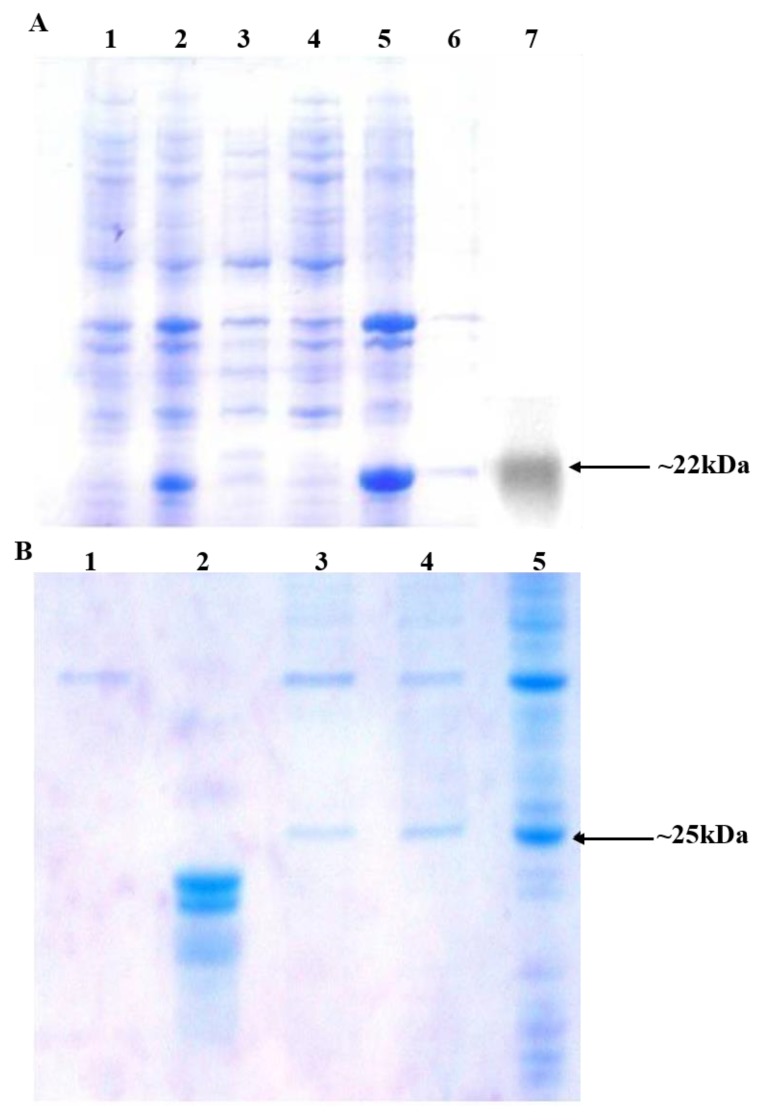
(**A**) Analysis of sub-cellular protein fractionations of *E. coli* harboring pOGH-2 by 15% SDS-PAGE and Western blotting. Lane 1: Un-induced sample; Lane 2: Total cell protein fraction from induced cells; Lane 3: Cytoplasmic fraction; Lane 4: Periplasmic fraction; Lane 5: Membrane fraction; Lane 6: Soluble fraction; Lane 7: Western blot of the purified rOGH-2 (Arrow indicates the position of rOGH at ≈22 kDa). (**B**) Analysis of sub-cellular protein fractionations of *E. coli* harboring pOGH-5 by 15% SDS-PAGE. Lane 1: Membrane fraction; Lane 2: Standard rOGH showing band at ≈22 kDa; Lane 3: Periplasmic fraction; Lane 4: Cytoplasmic fraction; Lane 5: Total cell protein fraction from induced cells; Arrow indicates the position of rOGH at ≈25 kDa.

**Figure 3:**
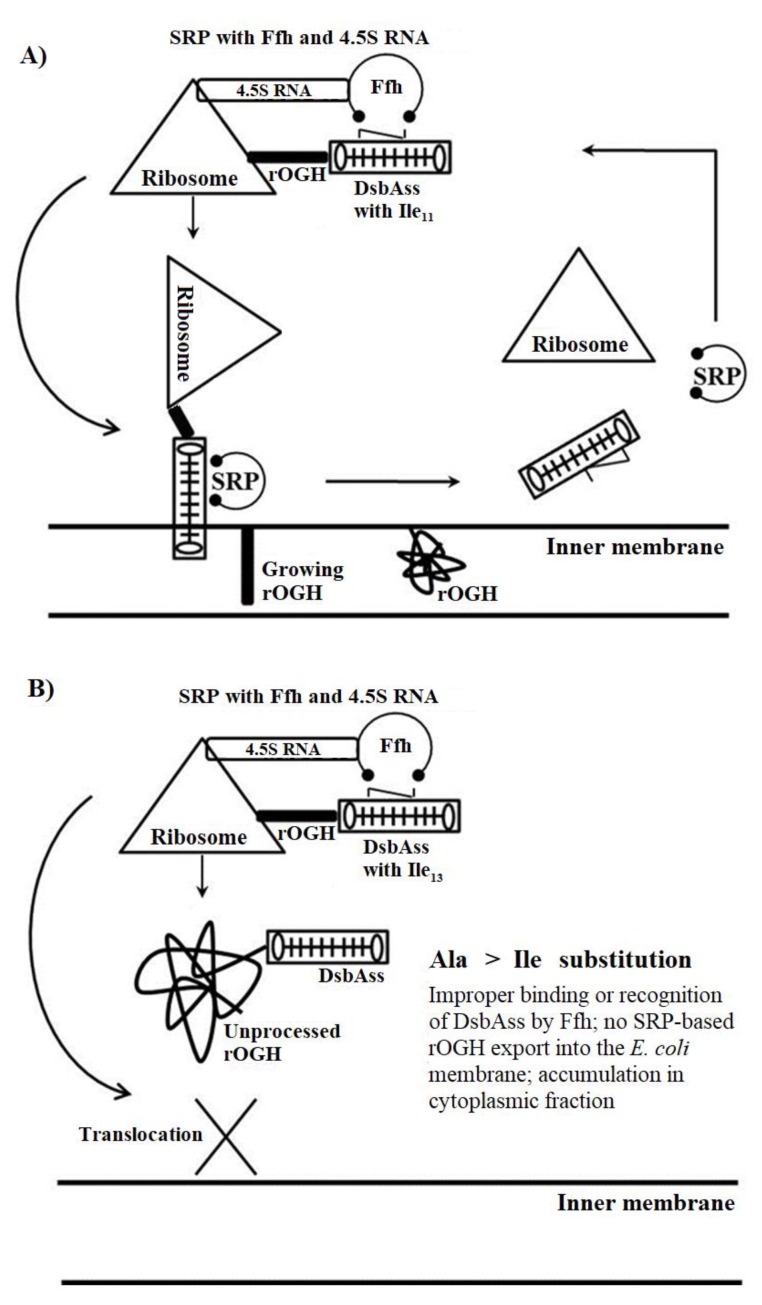
A model for signal repetition particle (SRP) routing of rOGH through DsbAss. (**A**) DsbAss with Ala_11_→Ile_11_ substitution; (**B**) DsbAss with Ala_13_→Ile_13_ substitution. Ffh: fifty-four homolog.

The comparison of efficiencies of rOGH export by all DsbAss variants showed different destinations of rOGH in the subcellular fractionations analysis. The normal destination of expressed rOGH with native DsbAss is reported to be the inner membrane of the cell [27]. The results of subcellular fractions of the constructs in SDS-PAGE showed that, together with the discrepancy in molecular mass of these two constructs (pOGH-2 and pOGH-5), the efficiency of rOGH export was also different. pOGH-2 acted like the native DsbAss and efficiently exported rOGH (100% of the protein was exported) to the inner membrane of *E. coli* (Figure 2A). However, pOGH-5 showed the presence of expressed rOGH in the cytoplasmic fraction, and no trace was found in the inner membrane (Figure 2B). These interesting findings of DsbA with the same hydrophobicity but difference in expression and destination of the desired protein led us to hypothesize that “the alanine at position -13 is crucial for the translocation of recombinant proteins in SRP mechanism”. The change of alanine at this position in the hydrophobic region of DsbAss induces conformational changes in the binding mechanism of the signal sequence and hampers the translocation of desired protein to the extracytoplasmic space of the cell (Figure 3). For further explanation of our model, molecular modeling analysis, with a detailed insight into these two variants, was carried out.

### 3.3. Molecular Modeling Analysis

In order to analyze the interaction between DsbAss variants and Ffh M-domain, a 28 amino acid finger loop was modeled using ab initio method implemented in MODELLER 9.15 to predict the conformation of the loop region. The multiple sequence alignment performed by HHpred between query sequence PDB ID: 1HQ1 against a database of HMMs representing proteins with known structures of the Ffh M-domain of different (e.g., PBD, SCOP) or annotated protein families was performed. Importantly, the Ffh M-domains of *B. subtilis* (PDB ID: 4UE4), *T. aquaticus* (PDB ID: 2FFH), and *M. jannaschii* (PDB ID: 4XCO) were the best hits for a multi-template for the disordered loop on E-value, query coverage, and identity. The program employed multi-templates and modeled the missing loop of Ffh M-domain with greater than 90% confidence. Such confidence reflected the validation of the core model having ≈2 Å RMSD from native protein structures. The finger loop in the reference structure of the *M. jannaschii* Ffh M-domain was also modeled using the same procedure.

**Figure 4:**
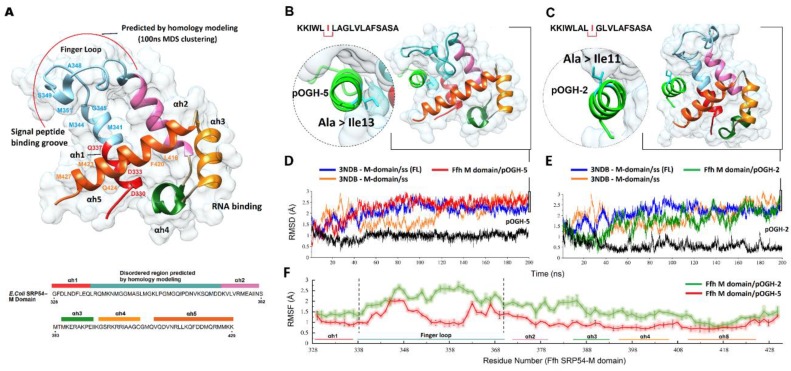
(**A**) The ribbon representation of the fifty-four homolog (Ffh) M-domain is enclosed by a molecular surface in white. The polypeptide chain is ramp-colored from red (N-terminal) to orange (C-terminal). The five alpha helices are rendered and colored accordingly (αh1—red, αh2—pink, αh3—green, αh4—golden, αh5—orange). The missing disordered region near the peptide binding groove is predicted by comparative homology modeling using HHpred and colored blue with the curved red line indicating the modeled portion. The two alpha helices (αh1 and αh5) and finger loop making up a signal peptide binding groove with key residues are labeled. The right panel shows the molecular dynamics simulated complexes of M-domain with pOGH-5 (**B**) and pOGH-2 (**C**) obtained after a 200 ns production run. This is followed by root mean square deviation (RMSD) trajectories in time-dependent manner (ns) of pOGH-5 (**D**) and pOGH-2 (**E**), with red and green color representing the M-domain and docked peptide in black. The RMSDs of the reference structure (3NDB) bound to signal sequence with (blue) or without the finger loop (FL) (orange) are also displayed. (**E**) Per-residue fluctuations of the M-domain throughout 200 ns are plotted for pOGH-5 (red) and pOGH-2 (green) along with the error bars calculated by comparing trajectories in different time frames, while the corresponding helices and finger loop regions are underlined with the same color code.

Considering the flexibility of finger loop, the model was subjected to 100 ns MD simulation for structure refinement and finger loop stability. A large ensemble of 1000 snapshots generated by an MD trajectory was used to select the representative conformation from the largest cluster based on RMSD cut-off of ≈1 Å as shown in Figure 4A. This representative conformation of Ffh M-domain was used to perform molecular docking studies with all signal peptide variants. The structural insight of the complete Ffh M-domain indicated five helices (αh1-5) whose structures and spatial conformations are quite identical to those in *B. subtilis, T. aquaticus*, and *M. jannaschii* [29,36,70]. The GTPase/M-domain linker (GM linker) together with perpendicular αh5, framed the base of the groove, while αh1 and the predicted finger loop (G328-D371) made up one side of the groove. Altogether, the structurally important segments were the GM linker, αh1, finger loop, and αh5, representing the hydrophobic groove which was about 12 Å narrow and 16 Å wide, covering an area of 192 Å. Within the binding groove, the hydrophobic property of groove was maintained by D330, D333, and Q337 of αh1, M341, M344, G345, A348, S349 and M351 of the finger loop, and L416, F420, M423, Q424, and M427 of αh5. As many as 11 of these 14 residues map onto the hydrophobic countenances of α helices. The side chain flexibility of these residues, including five methionine residues, were likely to enable the hydrophobic groove to bind with signal peptides of varying lengths [35] (Figure 4A). A further examination of the proposed signal peptide binding groove of the Ffh M-domain was conducted to check if it was of ample size and hydrophobicity to accommodate variable signal sequences.

In the current study, we attempted to predict the binding mode of DsbAss variants with the Ffh M-domain. We analyzed all eight DsbA constructs (pOGH-1 to -8) with this Ffh M-binding groove one by one. We found specific alteration of amino acids in the hydrophobic part of DsbAss that underwent changes in binding with the M-domain of Ffh with different binding affinities. These alterations were carefully analyzed in terms of binding energies and binding conformations of DsbAss. For molecular docking studies, two heuristic algorithms including ClusPro [57] and Patch dock [55] were employed to examine the constancy of interaction and to locate the conformation of signal peptides in hydrophobic groove of Ffh. For the docked poses generated by the Patch dock, the top 100 solutions were further rescored by Fire dock algorithm, which generated the best docking model of all 8 signal peptides. To generate high-quality complexes of a signal peptide with Ffh, the second highly efficient protein docking algorithm ClusPro was employed to elucidate favorable conformations of signal peptides. The predicted binding energies of DsbAss variants 1–8 highlighted the favorable lowest energy values of balanced, electrostatic, hydrophobic, and VdW+Elec (Supplementary Table S1).

Both signal peptides (pOGH-2 and pOGH-5) revealed same hydropathy indexes (1.539) but different expression size (≈22 and 25 kDa, respectively) (Table 2) which led us to explore a specific role of changing Ala to IIe in directing the conformational changes. To evidence this, docked conformations of the pOGH-2 and -5/Ffh M-domain were processed thorough the entire 200 ns MD simulation together with the reference structure (3NDB) and Cα. RMSD, and root mean square fluctuation (RMSF) trajectories were analyzed. RMSD clustering analysis was performed from the MD trajectory to find the most representative conformation with a cut-off value of ≈1 Å to explore the binding mode of peptides with the M-domain. The conformations with the lowest RMSD were selected from the largest cluster and are displayed in Figure 4B,C. Figure 4D,E shows the RMSD of the M-domain complexed with peptides. At over 50 ns, the MD trajectories of both M-domains-peptide complexes (pOGH-2 and -5) followed a similar trend. The Cα atom RMSD of M-domain/pOGH-5 showed slight deviations from 60 to 120 ns followed by a stable conformation for the last 80 ns with a <0.75 Å deviation. The RMSD of bound pOGH-5 remained stable throughout simulation after a small shift at 50 ns (Figure 4D), which indicated an important role in the conformational transition of Ile-3 inward the binding groove alongside the finger loop adjustment and remained stable as shown in Figure 4B. The RMSD of Ffh M-domain/pOGH-2 showed distinct deviations after 50 ns and pOGH-2 also showed instability throughout simulation (Figure 4D). Additionally, the finger loop, in this case, pulled to the other side as the simulation progressed in comparison to the pOGH-5 complex (Figure 4C). To further investigate the influence of finger loop on the M-domain, both reference structures, including the co-crystallized signal sequence with the *M. jannaschii* Ffh M-domain (3NDB, without finger loop) and modeled complex (with finger loop) with signal sequence revealed the stability of overall M-domain due to the presence of finger loop.

To analyze the mobility of each residue in M-domain around its average position with bound peptides, the RMSF values of all backbone atoms were calculated after 200 ns simulations. Based on the RMSF trajectories reflected in Figure 4F, the large fluctuations were observed in the finger loop (from Leu338 to Asp370) linking the αh1 and αh5. Among these, the high fluctuations with pOGH-2 may be related to the significant deviations of finger loop as compared to pOGH-5, which showed the stability of finger loop as shown in Figure 4D. The residues localized in other five helices have smaller fluctuations with <0.5 Å because of stable secondary structural elements.

**Figure 5:**
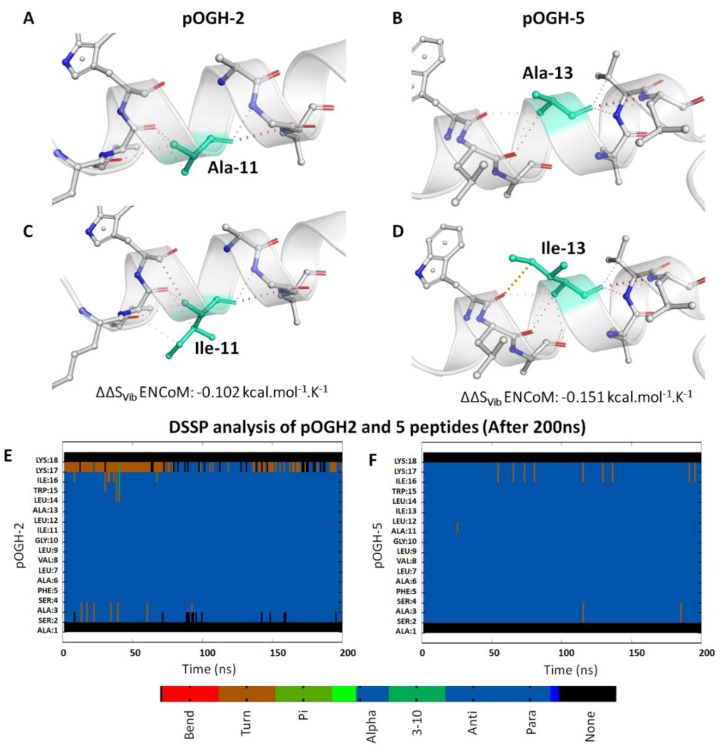
Representation of electrostatic and van der Waals interactions of native (**A**) and (**B**), and mutants (**C**) and (**D**) after replacement of isoleucine at positions −11 (pOGH-2) and −13 (pOGH-5). DSSP analysis for the secondary structure fluctuations throughout 200 ns for pOGH-2 (**E**) and pOGH-5 (**F**).

The helicity of both peptides was also determined using DSSP module of AMBER16, which revealed that the replacement of alanine to isoleucine retained helical conformation without diminishing their electrostatic and vdW interaction contributions in native (Figure 5A,B) and both mutant peptides (Figure 5C,D). The estimated vibrational entropy energy (ΔΔS_Vib_) and thermal stability (ΔΔG) from ENCoM server [71], which predicts the stability and dynamics upon mutations, also indicated decreased flexibility in pOGH-2 (ΔΔS_Vib_ ENCoM: −0.102 kcal mol^−1^ K^−1^; ΔΔG: 0.045 as stabilizing) and pOGH-5 (ΔΔS_Vib_ ENCoM: −0.151 kcal mol^−1^ K^−1^; ΔΔG: 0.012 as stabilizing), thus stabilizing the overall helical conformation. These thermodynamics predictions were found consistent with the secondary structure analysis using DSSP module of AMBER (Figure 5E,F), which provides clear evidence that the helicity of mutant peptides remained stable throughout 200 ns simulation period.

### 3.4. Binding Free Energy Calculations Using the MM-GBSA Method

To gain additional insight into the complex binding affinities, MM-GBSA analysis [72] was performed to calculate the total binding free energy (ΔG_tol_), separated by electrostatic (ΔE_ele_) van der Waals (ΔE_vdw_), and solute-solvent energies (ΔG_np_ and ΔG_p_) as summarized in Table 3. According to Table 3, the overall binding free energies (ΔG_tol_) of pOGH-2 and pOGH-5 complexes were −124.24 and −140.62 kcal mol^−1^. It should be noted that major total free energy contributions to the peptide binding were due to the favorable electrostatic interactions for both complexes pOGH-2 and pOGH-5 (ΔE_ele_ = −117.62 and −135.43 kcal mol^−1^) as compared to van der Waals interactions (ΔE_vdw_ = −87.36 and −99.48 kcal mol^−1^). Nonpolar solvation energies (ΔG_np_) also contributed towards peptide binding (ΔG_np_ = −14.85 and −15.41 kcal mol^−1^) while polar solvation energy (ΔG_p_) showed unfavorable contributions. The pOGH-5 has the highest ability to bind to M-domain as compared to pOGH-2 because of reasonably high electrostatic binding free energy which indicated a higher number of electrostatic and van der Waals interactions with the M-domain. The reference complexes of 3NDB-signal sequence with or without finger loop showed overall total binding energy as ΔG_tol_ = −127.41 and −106.88 kcal mol^−1^, respectively.

### 3.5. Per-Residue Decomposition Analysis Using MM-GBSA Method

**Figure 6:**
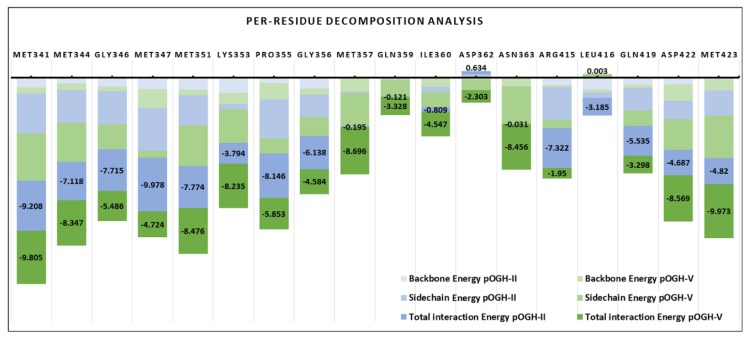
The stacking bar chart represented the binding free energy decomposition using the MM-GBSA method between the pOGH-2 and pOGH-5 complexed with the M-domain. Important residues for the M-domain binding groove are labeled on the top of each bar. The total interaction energies of key residues of binding groove with pOGH-2 (blue) and pOGH-5 (green) are displayed along with sidechain and backbone contributions with the same color gradient.

The M-domain binding groove residues that contributed to the peptide binding were further explored by per-residue decomposition analysis as illustrated in Figure 6. As examined, all methionine residues (M341, M344, M347, M351, Met357, and M423) of the binding groove contributed strongly with both peptides. A varying trend of total interaction energies was seen in finger loop (G328-D371) for both complexes in terms of backbone and side chain contributions. In the case of pOGH-5, the rise in free binding energy (ΔG_bind_ = −145.54 kcal mol^−1^) was obvious from the increased energy contributions of K353, M357, Q359, I360, D362, and N363, by −8.235, −8.696, −3.328, −4.547, −2.303, and −8.456 kcal mol^−1^, which decreased to −3.794, −0.195, −0.121, −0.809, 0.634, and −0.031 kcal mol^−1^ in pOGH-2, respectively. Such an increase in energy contributions was due to the structural adjustment of finger loop in a way, which positioned the sidechain conformation of some residues in favor of pOGH-5 binding as illustrated in sidechain energy percent in total interaction energy (Figure 6). Like M357, I360 and N363 completely oriented the sidechain position due to the more stable conformation of the pOGH-5–M-domain complex, as shown in the RMSD trajectory over 200 ns (Figure 4C,D). In contrast, these residues weakened or diminished interaction in pOGH-2 as shown in Figure 6. Interestingly, the D422 and M423 of α5 contributed significantly due to change to Ile at position -13 in pOGH-5, with total interaction energies of −8.569 and −9.973 kcal mol^−1^, respectively, values that were −3.882 and −5.153 kcal mol^−1^ higher than for pOGH-2, while a change of Ile to Ala at position -11 weakened the interactions due to unstable conformation of pOGH-2 (Figure 6). However, these important varying trends of interactions between key residues of finger loop and α5 might induce differences in the binding pose of peptides inside binding groove and overall free binding energies. The results illustrated that the change to Ile at positions -11 and -13 could disrupt some interactions between the binding groove residues and peptide. Further, pOGH-5 remained stable after a small shift at 50 ns, and structural adjustment of figure loop significantly enhanced the binding potential and hence could not translocate the desired rOGH into the inner membrane. We found rOGH at more than 60% in the cytoplasmic fraction and 20% in periplasmic fraction, and absent in the membrane fraction, while the complete translocation of rOGH was seen into the inner membrane in case of the pOGH-2 construct due to a lower number of interaction energetics (Figure 2A and Figure 5).

### 3.6. pOGH Construct Substitution with Lys → Arg at N Terminal of DsbAss

The two Lys residues of the N-terminal domain of DsbAss were substituted with Arg (pOGH-7 construct), as the presence of Arg in the N-region has previously been reported to be associated with better protein export [73]. Expression and translocation of recombinant expressed protein was analyzed by SDS-PAGE (Figure 1); the molecular mass of rOGH-7 was ≈22 kDa, and the protein was found in the membrane fraction, indicating the complete process of the DsbAss. However, the presence of Arg in the N-terminal domain does not seem to result in enhanced rOGH expression and/or its export into the *E. coli* inner membrane.

### 3.7. Influence of Substitution with Ser → Cys at C Terminal of DsbAss on rOGH Translocation

In pOGH-8 construct, two Ser residues present in the C-terminal domain of DsbAss were substituted with two Cys residues. The presence of Cys in the DsbAss has previously been described to be associated with the formation of disulfide bonds in the *E. coli* periplasm due to the oxidation–reduction role of DsbA [74]. In the present study, Ser → Cys substitution affected the translocation process of rOGH as analyzed by SDS-PAGE and was found in the cytoplasmic fraction with a molecular mass of ≈25 kDa (Figure 1). Apparently, this substitution changed the polar properties of C-terminal region, which affected the cleavage of DsbAss and led to the accumulation of an unprocessed, higher molecular mass rOGH-8 in the cytoplasmic fraction of *E. coli* cells.

## 4. Discussion

It is a well-established fact now that Dsb proteins are essential for the development of virulence of bacteria [5,6]. The DsbA catalyzes the disulfide bond formation in all the virulence factor proteins, i.e., toxins, adhesions, secretions, and motility, and in the absence of DsbA these proteins do not fold properly. The primary function of Dsb proteins is to help and protect the mature protein from improper oxidation [74,75].

The proteins destined for extra-cytoplasmic secretion are targeted to the translocon by SRP and secretory (Sec) pathways [18,23,24]. The DsbAss protein of specific interest directs export through SRP mechanism [8,23,76]. We designed a strategy to get a mutation in the DsbAss and observed its effects on the translocation of rOGH as a case study. The secretion of GH in periplasmic or extra-cytoplasmic space was earlier reported [19], where they used DsbAss for the periplasmic secretion of human GH. Recombinant GH is being studied in the cytoplasmic expression with the tiring refolding steps [77,78].

Moreover, the effect of signal peptide change was also studied on the expression and secretion of bovine GH, and no substantial effect was observed. In our previous work, we observed the effect of various signal sequences on the expression and secretion of OGH and eventually found the DsbAss as the best choice to produce biologically active rOGH in the extra-cytoplasmic space [27]. In this study, we studied the functional impact of a mutation in DsbAss on the translocation of rOGH. To our knowledge, this is the only study that describes the effect of individually mutated DsbAss on rOGH translocation in *E. coli*.

In the current study, we emphasized the substitution of amino acid in the hydrophobic (H) part of DsbAss as peptide helicity correlates with peptide hydrophobicity [79]. Also, it has been shown that in the H region of GspB signal sequence, glycine residue affects the routing of recombinant protein towards the Sec pathway [80]. Here we focused mainly on the Ala residues in the hydrophobic part of DsbAss. We changed each Ala in the H part with the most hydrophobic amino acid Ile and observed the effect of these alterations on the translocation of rOGH. From the subcellular fraction analysis of each construct pOGH-1 to -8 we found interesting configuration in the two Ala positions at -11 and -13 with respect to signal peptidase site in pOGH-2 and pOGH-5 constructs, respectively. The alteration of Ile in both constructs resulted in completely different translocation of rOGH; in the case of pOGH-2, the rOGH translocated to the inner membrane as reported previously with the native DsbAss [27], while pOGH-5 resulted in rOGH which mainly stuck in the cytoplasmic fraction. This finding led us to hypothesize the conformational change induced by specific Ile at -13, which altered the normal passage of translocation.

To confirm this hypothesis we performed molecular modeling analysis of these mutated constructs. In order to analyze the interaction between the DsbAss and Ffh M-domain, a 28 amino acid residue segment was modeled and underwent structural analysis. The finger loop of Ffh has been identified to consist of conformational variability that recommends its part in the binding and releases mechanism of signal sequences [33,36]. When the binding site is unoccupied, in order to make up for the hydrophobic signal sequence, the finger loop may change between conformations: open and closed. Thus, it potentially folds back into the groove that resides inside the Ffh crystal structure and is loaded with the finger loop of the nearby M-domain [33]. Over 200 ns MD simulations to analyze the conformational change, the most striking feature observed was the change in orientation of both DsbAss, where Ile of pOGH-5 was pointing inward (Figure 4B) and Ile of pOGH-2 was facing away from the groove (Figure 4C), which can eventually effect the whole mechanism of translocation.

Interestingly, the structural adjustment of the finger loop influenced the favorable conformation of pOGH-5 inside the binding groove (Figure 4B). Similarly, when compared to the reference structures, the incorporation of the finger loop enhanced the stability over co-crystalized 3NDB with bound signal sequence over 200 ns (Figure 4D,E). The MM-GBSA calculations further elucidated the total interaction energy differences upon conformational changes. The convergence of backbone RMSD of M-domain affirmed the stability of finger loop with pOGH-5 over time (Figure 4D) which anticipated high binding free energy (ΔG_tol_ = −140.62 kcal mol^−1^) as compared to pOGH-2 (ΔG_tol_ = −124.24 kcal mol^−1^) (Table 3). This high binding free energy was due to prominent electrostatic interactions which evidenced significant contributions of important finger loop residues (K353, M357, Q359, I360, D362, and N363), which were lacking in pOGH-2 (Figure 6). Moreover, two residues (D422 and M423) of α5 anticipated higher interaction energy (−8.569 and −9.973 kcal mol^−1^, respectively) with pOGH-5 (Ile-13), which remained stable over time alongside a favorable finger loop adjustment to maintain intact the pOGH-5 inside the binding groove. In reference complexes, the increase in overall total binding free energy (ΔG_tol_ = −127.41 kcal mol^−1^) was evident from the constant stability of 3NDB in the presence of finger loop which interacted more electrostatically as compared to the 3NDB complex without a finger loop (ΔG_tol_ = −93.88 kcal mol^−1^) (Table 3). The increased stability of the 3NDB-signal sequence complex lends further support the importance of finger loop towards SRP core function by providing domain stability [60]. The results of presented molecular modeling complement the experimental findings, i.e., pOGH-5 construct’s subcellular fractionation showed the presence of rOGH in cytoplasmic space rather than its destination inner membrane as reported in our previous results [27]. From these results, we hypothesized a model for SRP routing of the recombinant protein through the DsbAss, which means that a specific protein conformation in the hydrophobic part of the signal sequence affects binding with the M-domain of the Ffh. The Ala at position -13 in the H-domain of DsbAss was important for binding to the M-domain of Ffh in the SRP mechanism, and replacement by Ile-13 boosted the interaction which ultimately changed the orientation of H part of DsbAss. Since the conformation changes and regulates the latching of the signal sequence, there is a release of the heterodimeric domains of the SRP and its receptor, and a handover of the signal sequence to the translocon [81,82]. Therefore, we speculated that this change resulted in localization of rOGH primarily in the cytoplasmic fraction without cleavage of the signal peptide, as observed with the pOGH-2 constructs. To achieve biologically active rOGH, it should have disulfide bonds which are characteristic of GH protein. The mode of translocation (co-translational verse post-translational) can affect the folding process of a protein in the periplasm [23,74]. Therefore, it would be interesting to consider that the specificity of the amino acid is significantly important for a signal sequence in the SRP mechanism. Any defect in the signal sequence can lead to improper translocation of a protein [74] which can eventually conduct the formation of functionally impaired recombinant protein. We hypothesize that this strategy can be further used to combat bacterial virulence factors.

## Figures and Tables

**Table 1:** Sequence of primers used for the construction of pOGH plasmids -1 to -8. The underlined sequence represents DsbAss, whereas bold represents the OGH cDNA sequence and the non-underlined sequence is the site for *Nde*I restriction enzyme. Nucleotide variation incorporated in native DsbAss are underlined and bold.

Primer Name	Nucleotide Sequence
FP-1	5’-CAT ATG AAA AAG ATT TGG CTG GCG CTG GCT GGT TTA GTT TTA GCG TTT AGC GCA TCG GCG **GCC TTC CCA GCC ATG TCC**-3’
FP-2	5’-CAT ATG AAA AAG ATT TGG CTG **ATT** CTG GCT GGT TTA GTT TTA GCG TTT AGC GCA TCG GCG **GCC TTC CCA GCC ATG TCC**-3’
FP-3	5’-CAT ATG AAA AAG ATT TGG CTG **ATT** CTG **ATT** GGT TTA GTT TTA **ATT** TTT AGC **ATT** TCG GCG **GCC TTC CCA GCC ATG TCC**-3’
FP-4	5’-CAT ATG AAA AAG ATT TGG CTG **ATT** CTG **ATT** GGT TTA GTT TTA GCG TTT AGC GCA TCG GCG **GCC TTC CCA GCC ATG TCC**-3’
FP-5	5’-CAT ATG AAA AAG ATT TGG CTG GCG CTG GCT GGT TTA GTT TTA **ATT** TTT AGC GCA TCG GCG **GCC TTC CCA GCC ATG TCC**-3’
FP-6	5’-CAT ATG AAA AAG ATT TGG CTG GCG CTG GCT GGT TTA GTT TTA **ATT** TTT AGC **ATT** TCG GCG **GCC TTC CCA GCC ATG TCC**-3’
FP-7	5’-CAT ATG **AGAAGG** ATT TGG CTG GCG CTG GCT GGT TTA GTT TTA GCG TTT AGC GCA TCG GCG **GCC TTC CCA GCC ATG TCC**-3’
FP-8	5’-CAT ATG AAA AAG ATT TGG CTG GCG CTG GCT GGT TTA GTT TTA GCG TTT **TGT** GCA **TGT** GCG **GCC TTC CCA GCC ATG TCC**-3’

**Table 2:** Hydropathy indices of native and modified DsbA signal sequence (DsbAss) in pOGH constructs calculated using Swiss ExPASy ProtParam. The amino acid residues modified in native DsbAss are highlighted in bold.

pOGH Constructs	DsbA Signal Sequence	Description	Hydropathy Index	Approximate Size of Expressed rOGH (kDa)
−1	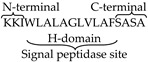	Native DsbAss	1.389	22
−2	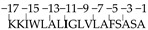	−11 Ala → Ile	1.539	22
−3	KKIWL**I**L**I**GLVL**I**FS**I**SA	−3, −6, −11 and −13 Ala → Ile	1.989	25
−4	KKIWL**I**L**I**GLVLAFSASA	−11 and −13 Ala → Ile	1.689	UD
−5	KKIWL**I**LAGLVLAFSASA	−13 Ala → Ile	1.539	25
−6	KKIWLALAGLVL**I**FS**I**SA	−3 and −6 Ala → Ile	1.689	22
−7	**RR**IWLALAGLVLAFSASA	−17 and −18 Lys → Arg	1.321	22
−8	KKIWLALAGLVLAF**C**A**C**A	−2 and −4 Ser → Cys	1.531	25

UD: Protein undetectable on the gel.

**Table 3:** Molecular mechanics generalized born surface area (MM-GBSA) binding free energy results for pOGH-2 and pOGH-5 (kcal mol^−1^) together with the reference structures with or without the finger loop.

Contributions (Total Binding Free Energy)	pOGH-2	pOGH-5	3NDB (without F-loop)	3NDB (with F-loop)
ΔE_ele_	−117.62	−135.43	−89.36	−118.44
ΔE_vdw_	−87.36	−99.48	−79.45	−95.01
ΔE_MM_	−204.98	−234.91	−168.81	−213.45
ΔG_p_	95.59	109.7	89.4	104.8
ΔG_np_	−14.85	−15.41	−14.47	−18.76
ΔG_sol_	80.74	94.29	74.93	86.04
ΔG_tol_	−124.24	−140.62	−93.88	−127.41

## Supplementary Materials

The following are available online at https://www.mdpi.com/2218-273X/9/4/133/s1, Table S1: Patchdock and ClusPro Cluster scores of native and mutated DsbA signal peptides.
