# Supplementary material for: Mutagenesis of DsbAss is Crucial for the Signal Recognition Particle Mechanism in Escherichia coli: Insights from Molecular Dynamics Simulations
Source: Biomolecules. 2019 Apr 3;9(4):133. doi: 10.3390/biom9040133 (PMC6523802; doi:10.3390/biom9040133)
Supplement: Supplementary file 1 [file biomolecules-09-00133-s001.pdf]

Supplementary Materials: Mutagenesis of DsbAss is Crucial for the Signal Recognition Particle Mechanism in *Escherichia coli*: Insights from Molecular Dynamics Simulations

Faiza Gul Durrani [1], Roquyya Gul [2,*], Muhammad Usman Mirza [3,4], Naheed Nazly Kaderbhai [5], Matheus Froeyen [3] and Mahjabeen Saleem [6]

1 School of Biological Sciences, Quaid-e-Azam Campus, University of the Punjab, Opp. Sheikh Zaid Hospital, Canal Bank Road, Lahore 54590, Pakistan; faiza_phd@hotmail.com
2 Faculty of Biological Sciences, Gulab Devi Educational Complex, Lahore, Ferozpur Road, Lahore 54000, Pakistan
3 Department of Pharmaceutical and Pharmacological Sciences, Rega Institute for Medical Research, Medicinal Chemistry, University of Leuven, Leuven B-3000, Belgium muhammadusman.mirza@kuleuven.be (M.U.M.); mathy.froeyen@rega.kuleuven.be (M.F.)
4 Center for Research in Molecular Medicine, The University of Lahore, Lahore 54000, Pakistan
5 Department Institute of Biological, Environmental and Rural Sciences, Aberystwyth University, Penglais, Aberystwyth, Ceredigion SY23 3FL, UK; nnk@aber.ac.uk
6 Institute of Biochemistry and Biotechnology, Quaid-e-Azam Campus, University of the Punjab, Opp. Sheikh Zaid Hospital, Canal Bank Road, Lahore 54590, Pakistan; mahjabeen.ibb@pu.edu.pk
* Correspondence: roquyya.gul@gdec.edu.pk; Tel.: +92-42-3591-804

Table S1. Patchdock and ClusPro Cluster scores of native and mutated DsbA signal peptides.

Signal peptides	Patchdock		Cluspro Cluster scores							
	Global Energy (Kcal/mol)	Attractive VdW	Balanced		Electrostatic-favored		Hydrophobic-favored		VdW + Elect	
			Center	Lowest energy	Center	Lowest Energy	Center	Lowest energy	Center	Lowest energy
**Native DsbA**	**-98.29**	-22.31	-1106.8	-1223.7	-1076	-1253.4	-1619.5	-1875.1	-169.1	-188.6
DsbA peptide 2	-101.93	-26.51	-1258.7	-1445	-1259.2	-1467.9	-2163.4	-2188.1	-165	-190.4
DsbA peptide 3	-109.59	-20.77	-1152.7	-1403.7	-1286.6	-1348.5	-1840.6	-2158.6	-141.9	-175.9
DsbA peptide 4	-90.16	-22.49	-1298.2	-1344.1	-1324.7	-1357.9	-1799.8	-2049	-151.7	-178.1
DsbA peptide 5	-96.2	-19.3	-1340.7	-1420.6	-1350.1	-1437.9	-1849.7	-2137.9	-155.8	-196.7
DsbA peptide 6	-95.48	-23.41	-1178.3	-1371.3	-1189.8	-1356.7	-1908.8	-2059.7	-147	-177.1
DsbA peptide 7	-102.25	-15.63	-1190.5	-1540.4	-1254.7	-1594.6	-2157.2	-2241.9	-150	-195
DsbA peptide 8	-95.54	-19.42	-1252.3	-1487.6	-1285.7	-1494.6	-1936.5	-2241.5	-157.6	-193.1
